# Limitations of the ARDS criteria during high-flow oxygen or non-invasive ventilation: evidence from critically ill COVID-19 patients

**DOI:** 10.1186/s13054-022-03933-1

**Published:** 2022-03-07

**Authors:** Michael Hultström, Ola Hellkvist, Lucian Covaciu, Filip Fredén, Robert Frithiof, Miklós Lipcsey, Gaetano Perchiazzi, Mariangela Pellegrini

**Affiliations:** 1 Anaesthesiology and Intensive Care Medicine, Department of Surgical Sciences, Uppsala University, Akademiska sjukhuset, ANOPIVA, Ing70, 2tr, 75185 Uppsala, Sweden; 2 Integrative Physiology, Department of Medical Cell Biology, Uppsala University, Uppsala, Sweden; 3 Hedenstierna Laboratory, Department of Surgical Sciences, Uppsala University, Uppsala, Sweden

**Keywords:** Acute respiratory distress syndrome, Mechanical ventilation, High-flow oxygen, Non-invasive ventilation

## Abstract

**Background:**

The ratio of partial pressure of arterial oxygen to inspired oxygen fraction (PaO_2_/F_I_O_2_) during invasive mechanical ventilation (MV) is used as criteria to grade the severity of respiratory failure in acute respiratory distress syndrome (ARDS). During the SARS-CoV2 pandemic, the use of PaO_2_/F_I_O_2_ ratio has been increasingly used in non-invasive respiratory support such as high-flow nasal cannula (HFNC) and non-invasive ventilation (NIV). The grading of hypoxemia in non-invasively ventilated patients is uncertain. The main hypothesis, investigated in this study, was that the PaO_2_/F_I_O_2_ ratio does not change when switching between MV, NIV and HFNC.

**Methods:**

We investigated respiratory function in critically ill patients with COVID-19 included in a single-center prospective observational study of patients admitted to the intensive care unit (ICU) at Uppsala University Hospital in Sweden. In a steady state condition, the PaO_2_/F_I_O_2_ ratio was recorded before and after any change between two of the studied respiratory support techniques (i.e., HFNC, NIV and MV).

**Results:**

A total of 148 patients were included in the present analysis. We find that any change in respiratory support from or to HFNC caused a significant change in PaO_2_/F_I_O_2_ ratio. Changes in respiratory support between NIV and MV did not show consistent change in PaO_2_/F_I_O_2_ ratio. In patients classified as mild to moderate ARDS during MV, the change from HFNC to MV showed a variable increase in PaO_2_/F_I_O_2_ ratio ranging between 52 and 140 mmHg (median of 127 mmHg). This made prediction of ARDS severity during MV from the apparent ARDS grade during HFNC impossible.

**Conclusions:**

HFNC is associated with lower PaO_2_/F_I_O_2_ ratio than either NIV or MV in the same patient, while NIV and MV provided similar PaO_2_/F_I_O_2_ and thus ARDS grade by Berlin definition. The large variation of PaO_2_/F_I_O_2_ ratio indicates that great caution should be used when estimating ARDS grade as a measure of pulmonary damage during HFNC.

## Introduction

The Berlin definition of the acute respiratory distress syndrome (ARDS)[1] is clinically useful as index of both organ dysfunction and severity of hypoxemia [2, 3]. There is growing interest in non-invasive respiratory support for acute hypoxemic respiratory failure [4]. The ongoing Corona Virus Infectious Disease 2019 (COVID-19) pandemic brought an even wider use of high-flow nasal cannula oxygen therapy (HFNC) and non-invasive ventilation (NIV) because of limited availability of mechanical ventilation (MV) [5]. While NIV provides a measurable positive end expiratory pressure (PEEP) that maybe consistent with the Berlin criteria, the level of PEEP generated by HFNC is uncertain [6, 7]. In addition, the inspiratory fraction of oxygen (F_I_O_2_) is uncertain because of leakage and variable dead space volume.

This means that the current ARDS criteria for grading hypoxemia are difficult or impossible to apply in non-invasively ventilated patients [8]. However, despite lacking clinical evidence, ARDS criteria have been applied to patients not undergoing invasive mechanical ventilation [9]. Recent reports have indicated some applicability of a low PaO_2_/F_I_O_2_ ratio during HFNC to predict mortality [10]. The main aim of the current study was to allow the accurate stratification of respiratory dysfunction during HFNC and NIV in COVID-19 ARDS patients by providing expected differences in PaO_2_/F_I_O_2_ ratio upon a change in ventilatory strategy.

## Materials and methods

PaO_2_/F_I_O_2_ ratios were investigated in critically ill adult patients with COVID-19 admitted to the intensive care unit (ICU) at Uppsala University Hospital in Sweden from March 14, 2020 until January 14, 202 and included in a prospective observational cohort study due to COVID-19. During the most intense part of the pandemic, HFNC was used in regular wards up to 60 L/min of 60% oxygen at our hospital. Reaching this level, patients were transferred to the ICU where increasing oxygen supplementation, prone position or a change of respiratory support would be performed. Actual flow rate was 50 ± 9 L/min at 66 ± 13% oxygen during step-up from HFNC, and 44 ± 10 L/min at 55 ± 13% oxygen during step-down to HFNC. Standard starting PEEP during NIV was between 5 and 10 cmH_2_O, and during MV 7 to 14 cmH_2_O (Table 1A and B). During step-down, a PEEP below 10 cmH_2_O during MV would be matched with a similar PEEP in NIV (Table 1A and B). The corresponding changes in PEEP are reported in Table 1C.

**Table 1:** Estimated positive end-expiratory pressure (PEEP) and change of PEEP during a change of ventilatory strategy

1st Strategy	2nd Strategy		
	HFNC	NIV	MV
*A: First strategy PEEP (cmH*_*2*_*O)*			
HFNC	–	3	3
NIV	6.3 ± 1.4	–	7.1 ± 2.1
MV	7.7 ± 2.1	7.1 ± 1.8	–
*B: Second strategy PEEP (cmH*_*2*_*O)*			
HFNC	–	5.8 ± 1.2	11.3 ± 4.0
NIV	3	–	13.1 ± 2.9
MV	3	6.5 ± 1.6	–
*C: Estimated change in PEEP (cmH*_*2*_*O)*			
HFNC	–	2.8 ± 1.2	8.3 ± 4.0
NIV	− 3.3 ± 1.4	–	6.0 ± 3.2
MV	− 4.7 ± 2.1	− 0.5 ± 2.0	-

The changes were from the first strategy to the second strategy that were either of high-flow nasal cannula (HFNC), non-invasive ventilation (NIV) or invasive mechanical ventilation (MV). The PEEP during HFNC was estimated to 3 cmH_2_O. **A**: PEEP before the change of strategy. **B**: PEEP after the change of strategy. **C**: Change in PEEP

We analyzed the PaO_2_/F_I_O_2_ ratio before a change in respiratory strategy compared to 30 to 60 min after a switch in respiratory support. Patients who were not in steady state ventilation, or who did not have a blood gas analysis within a reasonable time were excluded. For each included patient, the first two changes of respiratory support were used, resulting in six comparisons. (1) HFNC-to-NIV; (2) HFNC-to-MV; (3) NIV-to-HFNC; (4) NIV-to-MV; (5) MV-to-HFNC; (6) MV-to-NIV.

## Results

Two-hundred fifty-two changes of respiratory support in 148 patients were recorded. Median age was 66 (IQR = 55–73) years, 25% were women. Body mass index was 29 (25–33), and 26% of the included patients had a previous diagnosis of chronic pulmonary disease. Patients were admitted after 10 (8–12) days of symptoms with a simplified acute physiology score 3 (SAPS3) of 53 (47–59) and PaO_2_ /F_I_O_2_ ratio of 114 (101–148) mmHg.

**Fig. 1:**
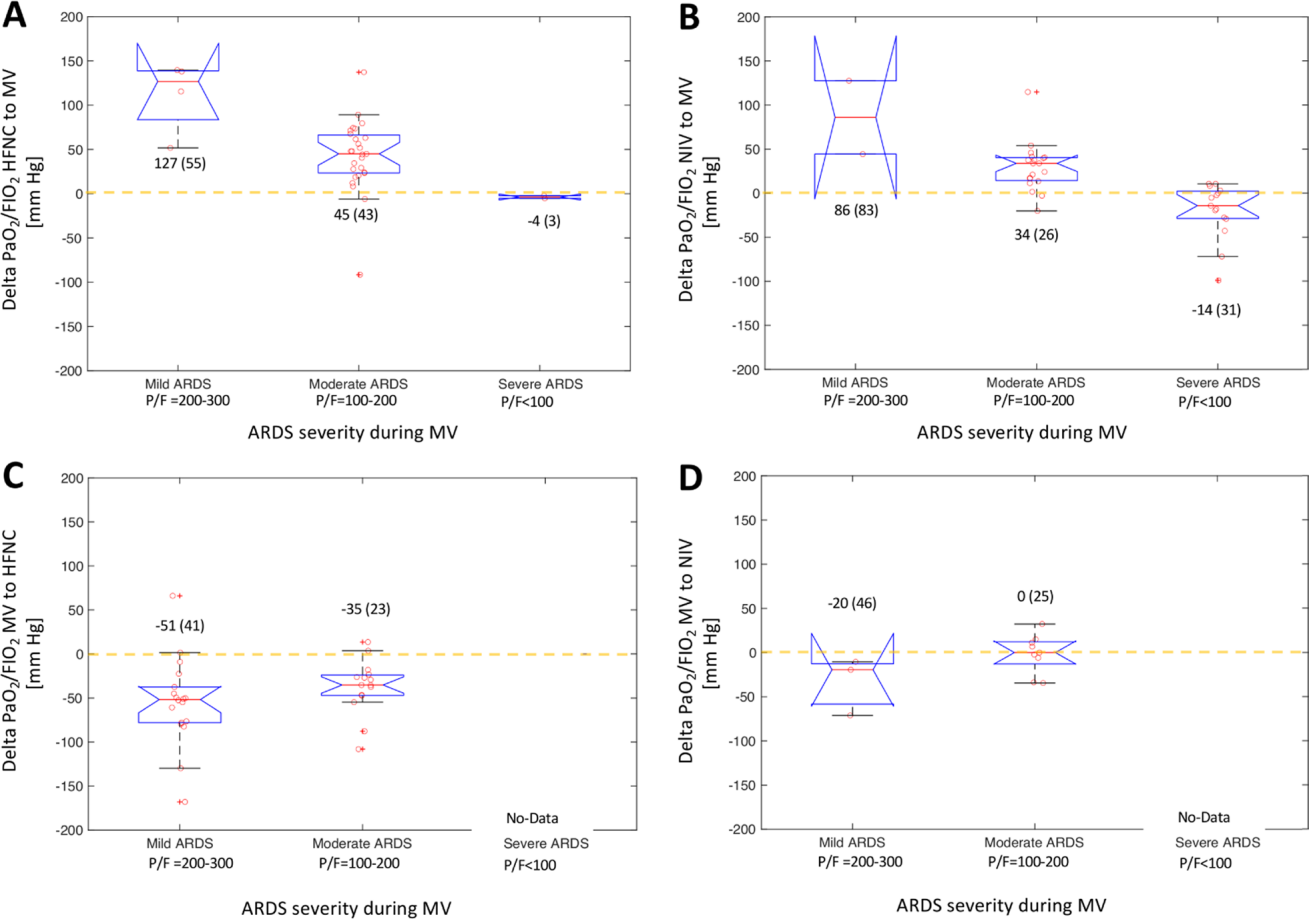
Change in PO_2_/F_I_O_2_ after a change in respiratory support in 148 critically ill COVID-19 patients grouped by ARDS severity during mechanical ventilation (MV) based on the Berlin definition. **A** HFNC to MV. **B** NIV to MV. **C** MV to HFNC. **D** MV to NIV

**Fig. 2:**
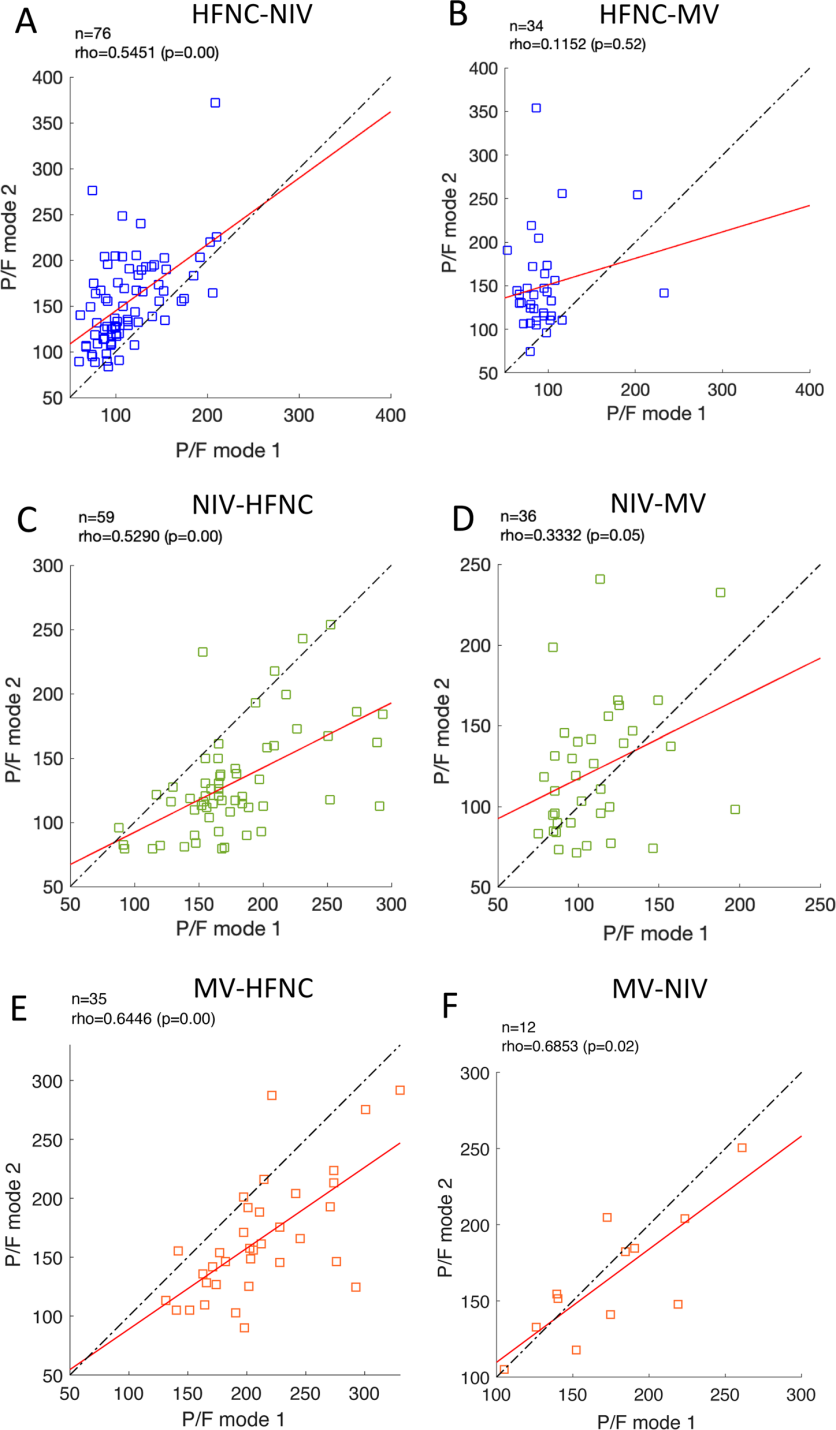
Correlation of PaO_2_/F_I_O_2_ ratio before and after a change in ventilatory support in 148 critically ill COVID-19 patients. The six tested groups were: **A** HFNC-to-NIV; **B** HFNC-to-MV; **C** NIV-to-HFNC; **D** NIV-to-MV; **E** MV-to-HFNC; **F** MV-to-NIV. Rho and P-value calculated using Spearman rank correlation

Changes to or from HFNC were biased to lower PaO_2_ /F_I_O_2_-ratio during HFNC, NIV-HFNC: -29(-15 to -60) mmHg, and HFNC-NIV: 41(21–65) mmHg, and more pronounced in HFNC-MV -45(-26 to -28) mmHg, and HFNC-MV 48(23–73) mmHg (Figs. 1A and C, 2A, B, C and E). On the other hand, changes between NIV and MV did not cause consistent differences in PaO_2_/F_I_O_2_ ratio NIV-MV: 11(− 7.5–38) mmHg and MV-NIV: − 4 (− 23—8) mmHg (Figs. 1B and D, 2D and F). All comparisons showed positive correlations between the strategies (Fig. 2 A, C, D, E and F), except HFNC-MV (*R*^2^ = 0.12, *P* = 0.52, Fig. 2B). The correlations improved during step-down from MV to NIV, or MV and NIV to HFNC (Fig. 2C, E and F).

**Fig. 3:**
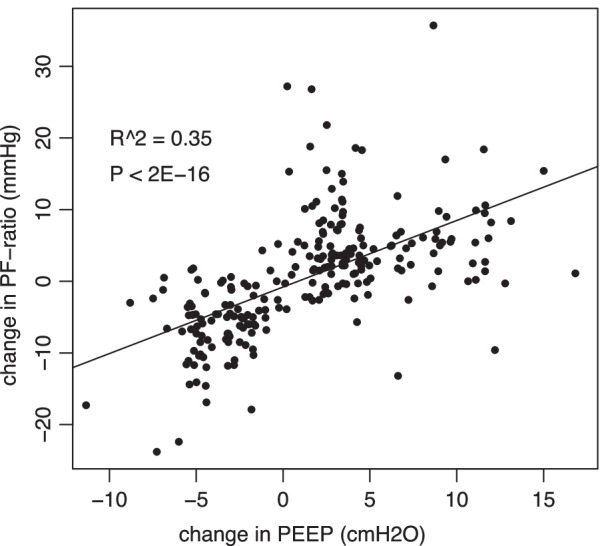
Correlation between the change in PO_2_/F_I_O_2_ ratio caused by a change of ventilatory strategy to the estimated change in positive end-expiratory pressure (PEEP) that accompanied that change. The changes were between high-flow nasal cannula (HFNC), non-invasive ventilation (NIV) and invasive mechanical ventilation (MV). The PEEP for HFNC was estimated at 3 cmH_2_O, while measured PEEP was used for NIV and MV. PEEP-values are jittered to avoid overlapping points in the graph. Pearson’s correlation was calculated using actual data

The effective delivery of PEEP is considered to be one of the most important differences between HFNC, NIV and MV, which is also an important determinant of the amount of open lung and thereby the PaO_2_ /F_I_O_2_ ratio. Therefore, we analyzed the correlation between the change in PaO_2_ /F_I_O_2_ and the change in PEEP for all individual strategy changes (Fig. 3). It is noteworthy that the correlation is good, but there is a large residual variation.

This resulted in reassignments between ARDS grades, showing general improvements in oxygenation during step-up from HFNC to NIV or MV. Mostly improvement during step-up from NIV to MV, although several patients showed worsened ARDS grade in this setting (Table 2A). Finally, worsened oxygenation, and increased ARDS grade was seen during step-down to HFNC from either MV or NIV, while step-down from MV to NIV only resulted in the reassignment of one patient in either direction (Table 2B).

**Table 2:** The number of patients who are reassigned to a new grade of acute respiratory distress syndrome (ARDS, Mild: 200–300 mmHg, Moderate: 100–200 mmHg, and Severe: < 100 mmHg) during a change in ventilatory strategy

1st Strategy	2nd Strategy		
	HFNC	NIV	MV
*A: Decreased ARDS grade*			
HFNC	–	42 (55%)	25 (74%)
NIV	1 (2%)	–	10 (28%)
MV	1 (3%)	1 (8%)	–
*B: Increased ARDS grade*			
HFNC	–	1 (1%)	1 (3%)
NIV	20 (34%)	–	5 (14%)
MV	14 (40%)	1 (8%)	–

The changes were from the first strategy to the second strategy that were either of high-flow nasal cannula (HFNC), non-invasive ventilation (NIV) or invasive mechanical ventilation (MV). Patients who decrease their ARDS grade, that improve in oxygenation are reported in **A**, and those who increase ARDS grade, or show worse oxygenation in **B**

## Discussion

The primary finding of this study is the high variability in the effect on PaO_2_/F_I_O_2_ ratio after changing ventilation strategy. Considering ARDS grade under MV as gold standard, PaO_2_/F_I_O_2_ ratio during HFNC could not reliably predict ARDS severity. The present findings may provide a rationale for using PaO_2_/F_I_O_2_ as ARDS criteria during NIV for decisions related to intubation or for prognosis but suggest great caution for its use during HFNC.

The main physiological reason for these findings is probably the efficiency of PEEP in counteracting alveolar collapse and concomitant shunt, as indicated by the excellent correlation between the change in estimated PEEP and the change in PaO_2_/F_I_O_2_ ratio. However, with a remaining variation of around 65% even the change in PEEP cannot reliably predict the result of a change in ventilatory strategy. Interestingly, PaO_2_/F_I_O_2_ ratio during step-down was markedly better, which probably indicates a well-recruited lung but also the fact that step-down happens when the lung has recovered to some degree. An additional factor which is well-known to affect PaO_2_/F_I_O_2_ ratio is inspired oxygen, which we have not controlled for [11]. This means that some of the difference in PaO_2_/F_I_O_2_ ratio is probably caused by changes in ventilation/perfusion mismatch caused by the change of respiratory strategy. While this could be controlled using a F_I_O_2_ of 1.0, this, in turn, would tend to increase absorption atelectasis and therefore the true shunt fraction especially in HFNC [12]. Further, not only the respiratory strategies used, but also the respiratory settings are known to affect PaO_2_/F_I_O_2_ ratio [13]. This means that the mandatory change from spontaneous ventilation to controlled ventilation at intubation will affect the results as well. The relationship between PaO_2_/F_I_O_2_ and F_I_O_2_ is influenced by multiple factors, such as the intrapulmonary shunt, arterio-venous difference of oxygen, partial pressure of arterial carbon dioxide, respiratory quotient and hemoglobin as well as the onset of absorption atelectasis [14]. The recognition of these physiological mechanisms as well as our results should prompt the development of more standardized procedures for grading hypoxemia and ARDS severity.

The current study had some limitations. The study focused exclusively on PaO_2_/F_I_O_2_ ratio as a measure of respiratory function, not taking into account the complexity and the multifactorial nature of hypoxic acute respiratory failure and of its management as, for instance, the use of prone positioning, the level of sedation, muscle relaxation or other pharmaceutical interventions. However, when referring to the PaO_2_/F_I_O_2_ ratio in our clinical practice as well as in the Berlin definition of ARDS [1], the PaO_2_/F_I_O_2_ ratio is used as an independent variable, not contextualized to, for instance, patients’ position, hemodynamic, muscle relaxation, sedation. Further, as a single center study, local clinical routines may influence the generalizability of the results.

In conclusion, PaO_2_/F_I_O_2_ ratio, in itself, is not sufficient to grade hypoxemia in ARDS patients during HFNC. PaO_2_/F_I_O_2_ ratio during NIV is a reasonable estimate for actual ARDS grade during MV. There are large individual variations in the effect of changes in ventilatory modality that suggests future ARDS definitions should treat respiratory support strategies separately. Importantly, our findings indicate that clinical trials investigating non-invasive respiratory support in ARDS patients can lead to misinterpretation of these patients’ outcomes. Additional prospective studies of PaO_2_/F_I_O_2_ ratio during HFNO will be needed to fully understand the relation of PaO_2_/F_I_O_2_ ratio to outcomes in patients with and without COVID-19.

## Data Availability

Data are available from the corresponding author on reasonable request (https://doi.org/10.17044/scilifelab.14229410).
